# A Systematic Review on Advances in Management of Oxidative Stress-Associated Cardiovascular Diseases

**DOI:** 10.3390/antiox13080923

**Published:** 2024-07-29

**Authors:** Soyeon Jin, Peter M. Kang

**Affiliations:** 1 Cardiovascular Institute, Beth Israel Deaconess Medical Center, Harvard Medical School, 3 Blackfan Circle, CLS 910, Boston, MA 02215, USA; 2 School of Pharmacy, Massachusetts College of Pharmacy and Health Sciences, Boston, MA 02115, USA

**Keywords:** reactive oxygen species, oxidative stress, cardiovascular diseases, antioxidant, vitamins, nanoparticles, biomarkers

## Abstract

Oxidative stress plays a significant role in the pathogenesis of cardiovascular diseases, such as myocardial ischemia/reperfusion injury, atherosclerosis, heart failure, and hypertension. This systematic review aims to integrate most relevant studies on oxidative stress management in cardiovascular diseases. We searched relevant literatures in the PubMed database using specific keywords. We put emphasis on those manuscripts that were published more recently and in higher impact journals. We reviewed a total of 200 articles. We examined current oxidative stress managements in cardiovascular diseases, including supplements like resveratrol, vitamins C and E, omega-3 fatty acids, flavonoids, and coenzyme-10, which have shown antioxidative properties and potential cardiovascular benefits. In addition, we reviewed the pharmacological treatments including newly discovered antioxidants and nanoparticles that show potential effects in targeting the specific oxidative stress pathways. Lastly, we examined biomarkers, such as soluble transferrin receptor, transthyretin, and cystatin C in evaluating antioxidant status and identifying cardiovascular risk. By addressing oxidative stress management and mechanisms, this paper emphasizes the importance of maintaining the balance between oxidants and antioxidants in the progression of cardiovascular diseases. This review paper is registered with the International Platform of Registered Systematic Review and Meta-analysis Protocols (INPLASY), registration # INPLASY202470064.

## 1. Introduction

Oxidative stress is commonly defined as the imbalance between oxidants and antioxidants, potentially leading to tissue damage [1]. This imbalance may stem from increased autoxidation of endogenous and exogenous compounds, such as reactive oxygen species (ROS) [2]. These ROS are typically produced during oxygen metabolism [3] or by environmental stressors, such as ultraviolet radiation, pollutants, heavy metals, and anticancer drugs [4,5]. If ROS accumulated and imbalance happens, the healthy antioxidant system usually tries to maintain ROS homeostasis by balancing their generation and elimination [6]. This is done through internal low molecular mass antioxidants (ascorbic acid, glutathione, tocopherols) and enzymes that regenerate antioxidants (superoxide dismutase (SOD), peroxidases, catalase) [7]. However, if ROS are excessive and not effectively managed by antioxidant mechanisms, this can cause oxidative stress that damages cellular components and significantly impacts cardiovascular health [8]. There are many articles indicating the importance of inhibiting oxidative stress in cardiovascular diseases. In this article, we will review the various notable therapeutic agents that are currently being used and the newer agents that have shown therapeutic potential to be used to mitigate and treat cardiovascular disease (CVD) associated with ROS. In addition, we will examine the biomarkers that can help identify the ROS-mediated CVDs.

## 2. Method

**Figure 1:**
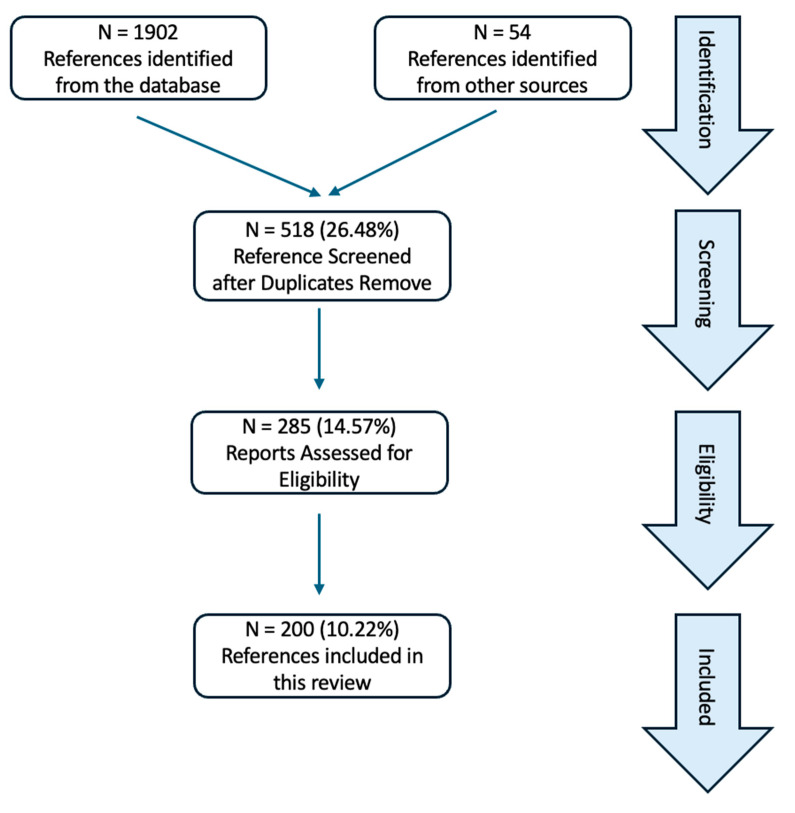
PRISMA flow diagram showing the study selection and identification.

For this systematic review, we searched relevant literatures in the PubMed database for studies or reviews published between database inception and 3 July 2024 utilizing specific keywords. The search terms employed were “oxidative stress”, “ROS”, “antioxidants”, and “cardiovascular disease”. We did not specify the date of the search, but we put emphasis on the more recent literatures published in the last few years. There were no exclusion criteria set for any specific journals, but we put emphasis on those manuscripts that were published in higher impact journals. This investigation aimed to identify and incorporate the most recent and pertinent studies, ensuring a robust foundation for the synthesis and analysis of information presented in the review. We followed the PRISMA guidelines for systematic reviews (Figure 1). This review paper is registered with International Platform of Registered Systematic Review and Meta-analysis Protocols (INPLASY), registration # INPLASY202470064

## 3. Oxidative Stress in Cardiovascular Diseases (Figure 2)

### 3.1. Oxidative Stress

Oxidative stress causes generation of ROS, such as superoxide (O_2_^•−^), hydrogen peroxide (H_2_O_2_), hydroxyl radical (^•^OH), ozone, and singlet oxygen [9]. These species are generated through cellular mechanisms and external sources, such as ultraviolet radiation, cigarette smoking, alcohol consumption, ingestion of nonsteroidal anti-inflammatory drugs (NSAIDs), infections, ischemia–reperfusion (I/R) injury, and various inflammatory processes [10]. Generally, O_2_^•−^ and H_2_O_2_ is formed in the process of oxygen (O_2_) reduction [11], primarily during the electron transport in mitochondria and from NADPH oxidase (NOX) enzymes, such as NOX4 [12]. H_2_O_2_ then undergoes a fast reaction when exposed to transition metals like iron, leading to the formation of hydroxyl radicals called Fenton reaction [13]. These hydroxyl radicals can initiate lipid peroxidation, leading to cellular membrane damage [14]. Additionally, H_2_O_2_ can react with other molecules like nitric oxide (^•^NO) to form peroxynitrite (ONOO^−^), which, along with hypochlorous acids (HOX), can cause tissue damage and inflammation, particularly related to the immune response of phagocytes [15].

**Figure 2:**
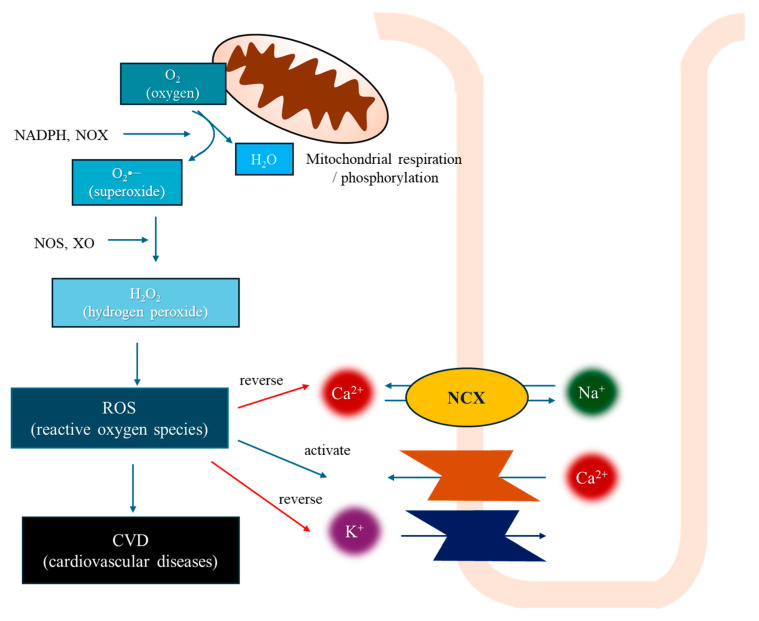
Oxidative stress in cardiovascular diseases. Cardiomyocyte electrophysiology is severely affected by increased ROS [16]. ROS reverse the function of the Na^+^/Ca^2+^ exchanger (NCX) and increase the influx of Ca^2+^ via the L-type calcium channels, leading to Ca^2+^ influx and Na^+^ efflux [17].

### 3.2. Sources of Oxidative Stress in the Cardiovascular System (Table 1)

Oxidative stress involves the regulation of mitochondria during oxidative phosphorylation [18]. Additionally, enzymes like NADPH oxidase, xanthine oxidase, and lipoxygenases contribute to homeostasis, potentially posing cardiovascular risks when dysregulated [19]. This overview provides the factors that cause oxidative stress and their potential implications for cellular and cardiovascular health.

**Table 1:** Sources of oxidative stress in the cardiovascular system.

Source	Role in ROS Production	Model Examined
NADPH Oxidase (NOX)	NOX generates superoxide radicals by transferring electrons from NADPH to oxygen, where dysregulation can contribute to oxidative stress and cardiovascular pathologies [20]. NOX1 in vascular smooth muscle cells and NOX2 in neutrophils and cardiovascular cells are generally harmful, while NOX4, broadly expressed in cardiovascular cells, might be protective, with NOX5 less implicated in pathology.	Human endothelial cells Human vascular smooth muscle cells
Xanthine Oxidase (XO)	XO catalyzes the conversion of hypoxanthine to xanthine and oxidation of xanthaine to uric acid, producing superoxide and hydrogen peroxide as byproducts, especially in ischemic injury and inflammatory responses in CVD [21].	Human endothelial cells Human cardiomyocytes
Nitric Oxide (NO)	NO causes peroxynitrite formation and highly reactive molecules, which contributes to endothelial dysfunction and cardiovascular disease under oxidative stress conditions [22].	Human endothelial cells
Lipoxygenases	Lipoxygenases catalyze oxidation of polyunsaturated fatty acids, producing lipid hydroperoxides, which contributes to inflammation and oxidative damage [23]. Increased lipoxygenase activity is linked to the progression of atherosclerosis and inflammation in human atherosclerotic lesions	Human aortic endothelial cells Mouse ApoE^−/−^ and Ldlr^−/−^ mouse models
Myeloperoxidase	Myeloperoxidase is released by neutrophils and generates hypochlorous acid, leading to oxidative stress and vascular function impairment [24].	Human plasma levels

### 3.3. Role of Oxidative Stress in the Pathogenesis of CVDs (Figure 3)

Oxidative stress can cause many different cardiovascular diseases. Cardiac muscles contain one of the highest densities of mitochondria and are heavily dependent on oxidative phosphorylation for proper function [24]. Throughout oxidative phosphorylation, cells generate ROS [25] that contribute to cardiac damage by affecting cellular components, such as lipids, proteins, and DNA. This leads to endothelial dysfunction, inflammation, and impaired vasomotor function [26]. The imbalance between ROS production and the body’s antioxidant defense mechanisms, often seen in conditions like atherosclerosis and hypertension, promotes oxidative stress [27]. Additionally, oxidative stress is implicated in the activation of signaling pathways associated with cardiac remodeling, hypertrophy, and apoptosis further exacerbating CVD progression [28,29]. Evidence also indicates that oxidative stress contributes to numerous cardiovascular conditions, including atherosclerosis, heart failure, cardiac arrhythmia, and ischemia–reperfusion injury [30]. For example, DNA damage induced by oxidative stress plays a crucial role in vascular remodeling following reperfusion injury [31]. Understanding the relationship between oxidative stress and cardiovascular health is vital to develop targeted treatments to reduce its harmful effects and prevent/manage heart diseases.

**Figure 3:**
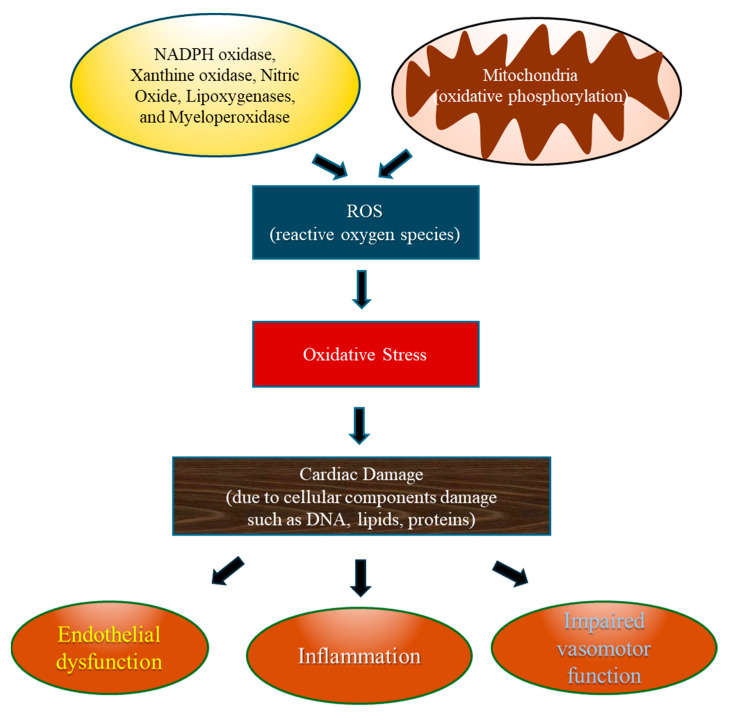
Oxidative stress in cardiovascular disease.

## 4. Cardiovascular Diseases Associated with Oxidative Stress

### 4.1. Myocardial Ischemia/Reperfusion Injury (MI/RI) and Oxidative Stress

Myocardial infarction (MI) is pathologically defined as myocardial cell death due to prolonged ischemia or myocardial ischemia/reperfusion injury (MI/RI) [32]. According to Forte et al., this reperfusion injury could be caused by different mechanisms, such as DNA damage. Persistent DNA damage presumably related to a temporary decreased expression of the DNA repair machinery and that of the antioxidant enzyme catalase may be playing a role [31]. In addition, ROS overexpression during reperfusion could lead to apoptosis, autophagy, and inflammation [33]. During reperfusion, ROS increase the level of calcium (Ca^2+^) [34] and proteins in the Bcl-2 family [35]. Increased Ca^2+^ and Bcl-2 related proteins could lead to the opening of the mitochondrial permeability transition pore, which in turn causes apoptosis of cardiac tissue [36]. ROS also activate the MAPK and NF-κB pathways, triggering external pathways of cell death, and induce apoptosis through endoplasmic reticulum stress [37]. Moreover, ROS provoke inflammatory responses causing heart damage. Reactive nitrogen species like NO are directly toxic to heart cells and could react with O_2_− to form damaging compounds, which could contribute to further myocardial damage [38]. The role of antioxidants are reducing myocardial ischemia/reperfusion injury by curbing oxidative stress and subsequent cell damage [39].

Platelets also play a pivotal role in myocardial infarction by promoting infarct expansion through oxidative stress, matrix metalloproteinase release, and platelet–leukocyte interactions [40]. Research also has shown the link between anti-platelets and oxidative stress in cardiovascular diseases, particularly during infarction and ischemia/reperfusion [41]. Treatments can also be focused on reducing mitochondrial dysfunction by regulating oxidative defense systems, such as nuclear factor erythroid 2-related factor 2 (Nrf2) and PI3K/Akt pathways, and Ca^2+^ overload [33]. Modulating autophagy and inflammatory responses also plays a crucial role in mitigating damage and improving cardiac function in MI/RI [42].

### 4.2. Atherosclerosis and Oxidative Stress (Figure 4)

Atherosclerosis is a chronic inflammatory disease caused by the accumulation of plaques inside arteries [43]. Atherosclerosis can begin with oxidative stress caused by the renin–angiotensin system (RAS), which could damage the blood vessel lining and causes endothelial dysfunction [44,45]. This leads to a series of events including endothelial and platelet activation, monocyte adhesion and transformation into proinflammatory macrophages, and increased uptake of oxidized low-density lipoprotein (ox-LDL) [46]. This process could lead to foam cell formation and worsening inflammation [47]. Evidence also supports that ox-LDL promotes lipid deposition within these plaques [48]. Studies showed that the elevated oxidized cholesteryl linoleate and HNE modified LDL levels in patients with atherosclerosis [49]. Additionally, isoprostanes and oxidized linoleic acid caused by oxidative stress also showed significant increases in atherosclerotic lesions compared to normal arteries [9].

Studies have shown that the renin–angiotensin–aldosterone system (RAAS) significantly impacts CVD, particularly atherosclerosis and hypertension [50,51]. In RAAS, angiotensin II (Ang-II) is generated from angiotensin I (Ang-I) by angiotensin converting enzyme (ACE). Then, angiotensin II activates angiotensin II type 1 receptor (AT1R), leading to two downstream effects. First, there is an increase in aldosterone secretion that increases low-density lipoprotein (LDL) and oxidized LDL (Ox-LDL). Second, there is NADPH oxidase stimulation, which produces ROS causing vasoconstriction [52]. Both pathways cause vasoconstriction, inflammation, and oxidative stress. These ultimately lead to atherosclerosis by the vascular endothelium damage and plaque formation.

Established therapies to treat oxidative stress-mediated atherosclerosis are statins, renin–angiotensin system inhibitors, and aspirin [53,54]. Studies have shown that statins decrease AT1R activity by inhibiting the transcription factor NF-κB signaling, which is essential for Ang II-mediated activation of AT1R [55]. For example, Kiaie et al. showed that statins regulate the AT1R pathway by lowering LDL, with a linear relationship between oxidized LDL levels and AT1R expression in various cells. These findings suggest that the anti-inflammatory effects of statins contribute to their ability to regulate AT1R activity by targeting NF-κB signaling pathways. In addition, Mansouri et al. demonstrated that statins achieve their antioxidant activity by suppressing the inflammatory reactions via their effects on Nrf2 pathway, leading to inhibition of ROS production [56].

Antioxidants, such as glutathione and uric acid, are synthesized in our body [57]. However, vitamins E and C, flavonoids, and carotenoids can be obtained from the diet [58]. Current research studies are also exploring novel approaches by targeting oxidative stress in atherosclerosis, such as mitochondrial ROS, nanotechnology-based drug delivery, gene therapies, and anti-miRNAs [59]. Other studies show that spironolactone could protect against endothelial dysfunction by reversing the effects of aldosterone-induced oxidative stress on eNOS expression [60]. SiRNA drugs currently in development can potentially reduce LDL-C and Lp(a) levels with infrequent dosing; phase III trials are in progress [61].

**Figure 4:**
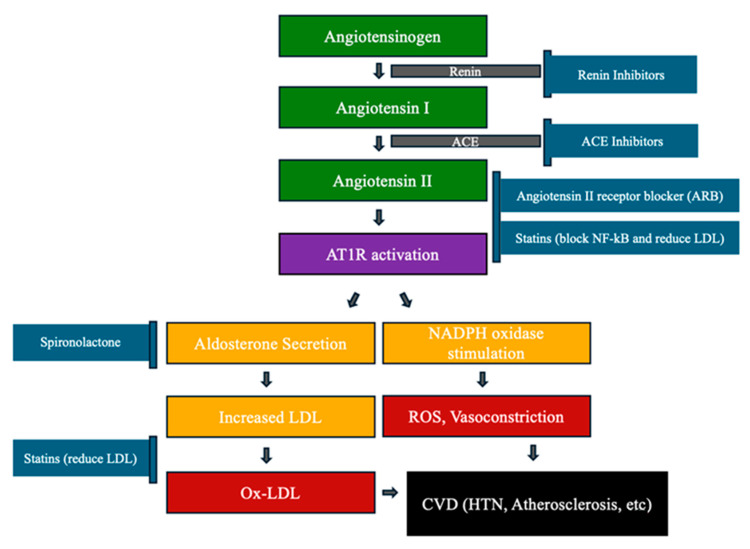
Statin/renin–angiotensin system inhibitors in atherosclerosis.

### 4.3. Heart Failure (HF) and Oxidative Stress

Heart failure is a complex clinical syndrome that can result from any structural or functional cardiac disorder that impairs the ability of the ventricle to fill with or eject blood [62]. Heart failure can cause fluid retention, leading to pulmonary congestion and peripheral edema, as well as reduced cardiac output [63]. The oxidative stress, characterized by the excessive ROS production, also has been shown to impact heart and blood vessels, causing heart failure [28].

ROS can directly impact contractile function, activate hypertrophy signaling pathways, induce apoptosis, promote cardiac fibroblast proliferation, and trigger extracellular matrix remodeling [64]. However, these cellular processes are key players in the development and progression of maladaptive myocardial remodeling and HF [28].

Multiple clinical trials have supported the efficacy of three beta-blockers (carvedilol, metoprolol, and bisoprolol) in the treatment of heart failure [65]. Particularly, carvedilol is demonstrated to promote the endothelial NO production, to reduce NO inactivation, and to protect against ROS through scavenging free radicals and suppressing their generation [66]. Other studies also demonstrated that bisoprolol prevents the progression of cardiac dysfunction in a dilated cardiomyopathy model by mitigating oxidative stress and inflammation [67].

### 4.4. Hypertension (HTN) and Oxidative Stress

HTN is caused by genetic, environmental, and pathophysiologic factors, leading to vascular dysfunction, cardiovascular remodeling, renal dysfunction, and sympathetic nervous system stimulation [68]. Studies have shown that oxidative stress is a key factor, which leads to endothelial dysfunction, aldosterone release, and inflammation; all these factors contribute to elevated blood pressure [69,70]. Increased oxidative stress may damage the endothelium, impair endothelium-dependent vascular relaxation, and increase vascular contractile activity [71]. This imbalanced activates the immune system and further promotes cytokines and chemokines release, which promotes oxidative stress and ROS production [72].

In addition, various antioxidants have been shown to reduce oxidative stress and blood pressure by interrupting free radical chain reactions and improving endothelial function [73]. The study by Craighead et al. investigated the effectiveness of high-resistance inspiratory muscle strength training (IMST) in lowering blood pressure and improving endothelial function among midlife and older adults with above-normal systolic blood pressure [74]. IMST improved endothelial function significantly, which was linked to increased nitric oxide bioavailability, greater activation of endothelial nitric oxide synthase, and reduced oxidative stress [74]. Studies have shown that inactive oxidized protein phosphatases, which are important for removing phosphates from signaling molecules, could also cause hypertension by affecting other proteins downstream [68,75].

## 5. Antioxidant Supplements in CVD (Table 2)

Antioxidant supplements play a significant role in the prevention and treatment of CVD by mitigating oxidative stress. This section will review the current use of antioxidant supplements for treating oxidative stress induced CVDs. These molecules were selected due to their proven efficacy in mitigating oxidative stress and enhancing cardiovascular health. Their effects are also supported by the extensive research on their underlying mechanisms and therapeutic outcomes in both experimental and clinical settings.

**Table 2:** Antioxidant supplements for cardiovascular diseases.

Antioxidant Supplement	Description
Resveratrol	- A natural polyphenolic compound - ↓ ferroptosis and the USP19/Beclin1-mediated autophagy pathway - ↑ the Nrf2 pathway
Vitamins C, D, and E	- Scavenge free radicals, ↓ oxidative damage, regulate RAAS activity - ↓ parathyroid hormone levels.
Omega-3 (EPA and DHA)	- ↑ antioxidant molecule via upregulation (↑) of NRF2 pathway - ↑ glutathione peroxidase (GPx) and superoxide dismutase (SOD) - ↓ levels of malondialdehyde (MDA)
Flavanoids	- ↓ xanthine oxidase activity - ↓ platelet adhesion, ↑ endothelial function by vasodilation
Coenzyme Q-10	- ↑ antioxidant production, ↓ lipid peroxidation - Protect blood vessels by preserving NO
Curcumin	- ↓ COX-2, LOX, NF-kB, and iNOS - ↓ inflammatory markers like CRP, TNF-α, and IL-6

### 5.1. Resveratrol

Resveratrol, a natural polyphenolic compound found in various fruits and plants, is investigated in the context of myocardial I/R injury [76]. Numerous studies have explored the antioxidant effects of resveratrol on I/R injury and the potential mechanisms involving ferroptosis and lipid peroxidation [77]. Studies showed that resveratrol attenuates oxidative stress and reduces ferroptosis in myocardial I/R injury [78,79]. Specifically, resveratrol treatment leads to a decrease in malondialdehyde (MDA) levels [80] and increase in superoxide dismutase activity. It also modulates the expression of ferroptosis-related proteins [81], including glutathione peroxidase 4 and ferritin heavy chain 1, in both in vivo and in vitro models [82]. Resveratrol also modulates the ubiquitin-specific peptidase 19 (USP19)/Beclin1-mediated autophagy pathway, which is implicated in the regulation of ferroptosis [83,84]. Additionally, resveratrol has shown potential to protect and to improve cardiovascular function by reducing oxidative stress and inflammation through the activation of the Nrf2 pathway in various experimental models [85]. The Nrf2 transcriptional pathway regulates the production of proteins essential for the management of neutrophils and ROS [86]. These studies demonstrated the potential use of resveratrol as a therapeutic agent for the prevention and treatment of cardiovascular diseases associated with MI/RI [87].

### 5.2. Vitamins

Vitamins, particularly Vitamin C, D, and E, serve as essential antioxidants in the prevention of oxidative stress-mediated CVD [88]. Many studies demonstrated the role of vitamins as potent antioxidants, as they scavenge free radicals and reduce oxidative damage [89,90]. They also have been shown to regulate RAAS activity and lower parathyroid hormone levels [91,92]. Shite et al. showed that vitamins can alleviate tissue oxidative stress in congestive heart failure (CHF), potentially improving cardiac function by reducing beta-receptor downregulation and mitigating sympathetic nerve terminal abnormalities [93]. This is also supported by Ellis et al., who studied 55 CHF patients and confirmed that vitamin C reduces oxidative stress and decreases neutrophil O_2_− generation with long-term use [94]. Raygan et al. conducted a double-blind trial with 60 diabetic coronary heart disease patients for 12 weeks, receiving vitamin D and probiotics. They showed significant mental and metabolic health improvements and beneficial effects in plasma NO and plasma total antioxidant capacity [95]. Mirmiran et al. also found that higher vitamin E intake was inversely associated with CVD incidence, highlighting its potential protective role in preventing CVD events [96]. Incorporating a diet rich in fruits, vegetables, and whole grains, which are abundant sources of these vitamins, can contribute to a reduced risk of oxidative stress-mediated cardiovascular diseases [97].

### 5.3. Omega-3

Omega-3 (n-3) fatty acids are abundant in foods and dietary supplements, such as fish oil [98]. They are polyunsaturated fats with eicosapentaenoic acid (EPA; 20:5n-3) and docosahexaenoic acid (DHA; 22:6n-3) [98]. Numerous randomized, controlled trials have investigated the effects of EPA and DHA in patients with existing CVD [99,100]. For example, Alexdanter et al. conducted meta-analysis of prospective cohort studies and found a significant reduction in any CVD event risk with higher EPA+DHA intakes [101]. Sakai et al. found that EPA and DHA significantly decreased DNA damage markers and ROS, while increasing antioxidant molecule levels through the upregulation of the Nrf2 pathway. These findings suggest that EPA and DHA have protective role against CVD by enhancing cellular antioxidant responses [102]. A meta-analysis of 38 trials conducted by Khan et al. with 149,051 people found that omega-3 fatty acids reduced cardiovascular mortality and improved outcomes [103]. Interestingly, EPA monotherapy has shown better effects than EPA + DPA but higher risk of bleeding and atrial fibrillation. Meta-analysis done by Heshmati et al. showed that omega-3 fatty acids supplementation significantly increased the activities of glutathione peroxidase (GPx) and SOD, which are crucial enzymes in the antioxidant defense system [104]. Heshmati et al. also demonstrated that the omega-3 supplementation significantly decreased levels of malondialdehyde, a marker of oxidative stress [104]. In summary, EPA and DHA have shown to have beneficial effect in preventing CVD and their cardioprotective mechanisms have been elucidated [105,106,107].

### 5.4. Flavanoids

Flavonoids, abundant in fruits and plants, are diverse secondary metabolites comprising a benzopyrone ring with phenolic groups [108]. Flavonoids lower ROS and nitrogen species levels by donating electrons to radicals like peroxynitrite, hydroxyl, and peroxyl, forming stable flavonoid radicals and stabilizing these radicals [109]. Also, flavonoids can effectively reduce xanthine oxidase activity [110]. These antioxidant properties of flavonoids in preventing cardiovascular diseases have been demonstrated in many studies [108,111], especially in their antiplatelet effect and antihypertensive effects [109,112].

Additionally, the ability of flavonoids to interfere with lipid metabolism, decrease platelet adhesion, and improve endothelial function by vasodilation has also been demonstrated in multiple studies [113,114,115,116]. Sapian et al. reviewed how flavonoids can alleviate diabetic cardiomyopathy by targeting oxidative stress induced by mitochondrial dysfunction, showcasing their potential role as therapeutic agents due to their antioxidant properties [117].

### 5.5. CoEnzyme Q-10 (CoQ10)

Coenzyme Q10 (CoQ10) or ubiquinone is a fat-soluble molecule essential for electron transfer in mitochondria and ATP production, which enhances antioxidants and reduces oxidative stress and lipid peroxidation [118]. CoQ10 can also increase antioxidant production, reduce oxidative stress in hypertensive patients, lower lipid peroxidation, and protect blood vessels by preserving NO [119]. According to Sharifi-Rad et al., CoQ10, in its active form (quinol), may scavenge several ROS and regenerate other damaged antioxidants (including Vitamin C and E) [120]. Mortensen et al. conducted a multicenter randomized trial with 420 patients by giving them CoQ10 or a placebo alongside standard treatment over two years. They concluded that long-term adjunctive therapy is safe, improves symptoms, and reduces major adverse cardiovascular events in patients with chronic HF [121]. In terms of dosing, the meta-analysis conducted by Rabanal-Ruiz et al. suggested that prolonged CoQ10 supplementation at doses of 200 mg/day or higher is safe and reduces oxidative stress, leading to cardiovascular mortality [122]. There are many discussions on the dose of CoQ10, from 90 mg to 400 mg daily, which depends on the severity of the disease [122,123,124,125,126]. CoQ10 has shown benefits in treating CVD, but the consensus on optimal dosing across other diseases and formulations in clinical use are still controversial [127].

### 5.6. Curcumin

Curcumin is one of the components of turmeric and possesses anti-inflammatory and antioxidative features [128]. Its anti-inflammatory effects are studied by inhibiting enzymes like COX-2, LOX, NF-kB, and iNOS, leading to antioxidative properties [129,130]. This is supported by the study of Dehzad et al., which concluded that turmeric/curcumin supplementation significantly reduces inflammatory markers like CRP, TNF-α, and IL-6, while enhancing antioxidant activity, suggesting its potential as an intervention for improving inflammatory and oxidative status [131].

## 6. Pharmacological Management of Oxidative Stress-Medicated Cardiovascular Diseases (Table 3)

### 6.1. Antioxidants

Oxidative stress plays a significant role in the pathogenesis of various CVDs. Addressing oxidative stress through pharmacological interventions can potentially mitigate myocardial damage and improve patient outcomes. In this section, several antioxidants are discussed that have been shown to have cardioprotective effects in clinical settings.

**Table 3:** Pharmacological management of oxidative stress-medicated CVD.

1. Pharmacological Antioxidants	
N-acetyl-cysteine (NAC)	- A synthetic derivative of L-cysteine - Mitigates cardio-renal syndrome type 3
Puerarin	- Target AMPK-mediated ferroptosis signaling,
Melatonin	- Reduce oxidative stress and improving vascular function.
Irisin	- Reducing oxidative stress and apoptosis
Cannabinoids	- Interact with the endocannabinoid system
2. Nanoparticles	
PVAX	- Nanoparticles containing vanillyl alcohol - Reduce ROS, inflammation, and apoptosis
Curcumin Nanomicelle	- ↓ COX-2, LOX, NF-kB, and iNOS - ↓ inflammatory markers like CRP, TNF-α, and IL-6

#### 6.1.1. N-Acetyl-Cysteine (NAC)

NAC is a synthetic derivative of the endogenous amino acid L-cysteine and a precursor of GSH [132]. Reyes et al. showed that NAC decreases protein expression linked to stress pathways, mitigates myocardial fibrosis, and lessens right ventricular hypertrophy, while also restoring glutathione levels, reducing oxidative stress, and improving MAPK signaling in aortic stenosis rats [133]. NAC has direct antioxidant effects and inhibits the inflammatory response by blocking NF-κB, a key regulator in inflammation and immune reactions to oxidative stress [134]. Additionally, a randomized, double-blind NACIAM (N-acetylcysteine in acute myocardial infarction) trial investigating the use of high-dose NAC (20 mg/min in the first hour followed by 10 mg/min for the remaining 47 h) alongside low-dose nitroglycerin found that NAC significantly reduced infarct size and increased myocardial salvage compared to placebo [135]. Also, Tossios et al. studied 40 patients undergoing coronary artery surgery who were randomized to receive either NAC or a placebo during their procedure. They observed a significant reduction in oxidative stress markers, such as 8-iso-prostaglandin-F(2) alpha and nitrotyrosine, suggesting that NAC may effectively reduce myocardial oxidative stress during cardiac surgeries [136].

#### 6.1.2. Puerarin

Puerarin, an isoflavone derived from the *Radix Pueraria*, has been studied for its protective effects against sepsis-induced myocardial injury through AMP-activated protein kinase (AMPK)-mediated ferroptosis signaling [137]. The studies have suggested that targeting AMPK or key proteins involved in ferroptosis could be a viable prevention and treatment of lipopolysaccharide (LPS)-induced myocardial injury [138]. Puerarin also exerts significant cardioprotective effects in various settings. It has shown to inhibit LPS-induced myocardial injury and cardiac dysfunction. In addition, it inhibits ferroptosis, a regulated cell death process associated with iron and lipid peroxidation through the up-regulation of AMPK phosphorylation [139]. A clinical trial demonstrated that puerarin significantly reduces markers of myocardial injury and improves cardiac function, supporting its potential therapeutic use in humans [140]. Zhou et al. examined the effect of puerarin treatment in human bronchial epithelial cells (HBECs) exposed to cigarette smoke extract. Puerarin activated the PI3K/AKT/mTOR signaling pathway [137]. This suggests puerarin exerts its effect by modulating ferroptosis-related proteins and preventing iron accumulation in myocardial tissues, which contributes to its protective effect against myocardial injury [140].

#### 6.1.3. Melatonin

Melatonin is one of the physiological antioxidants, exerting its ROS scavenging function in mitochondria. In the USA, melatonin is considered as a supplement, but in other countries (e.g., Europe or Asia), melatonin is considered a pharmaceutical compound. Also, melatonin has been shown to have beneficial effects in ischemic heart disease and prevents ischemia reperfusion-mediated myocardial damage [141]. Franco et al. explored the effects of low-dose melatonin supplementation on oxidative stress and vascular health in patients with essential hypertension [142]. They showed that melatonin supplementation in patients with essential hypertension significantly improved arterial stiffness and reduced total oxidative stress level, suggesting the potential benefits of melatonin in improving vascular function and reducing oxidative stress in hypertensive patients [142]. The association between low melatonin secretion levels and a higher risk of myocardial infarction has also been suggested [143], supporting the role of endogenous melatonin in cardiovascular pathologies [144]. Chitimus et al. also showed that supplementation with melatonin could improve adult cardiovascular homeostasis [145]. Melatonin binds to the catalytic site of the cytosolic enzyme quinone oxidoreductase 2 (QR2), also known as MT3 receptor, regulating QR2 function to reduce ROS production [146]. This detoxification process by melatonin has been shown to play a crucial role in maintaining cellular redox homeostasis, protecting against oxidative stress, neurodegeneration, and cardiovascular diseases [147].

#### 6.1.4. Irisin

Irisin, a hormone-like myokine, is cleaved and secreted from fibronectin type III domain-containing protein 5 (FNDC5), and is induced by aerobic exercise [148]. As a pro-myogenic factor, Irisin could induce skeletal muscle hypertrophy, activate satellite cells, enhance protein synthesis, reduce protein degradation, promote mitochondrial biogenesis and rescue the loss of skeletal muscle mass [149]. In a study exploring skeletal muscle changes in heart failure due to myocardial infarction, the impact of Irisin, a muscle-secreted hormone, was examined in the context of oxidative stress and cell apoptosis [150]. Utilizing myocardial infarction models along with aerobic exercise in fibronectin type III domain-containing protein 5 knockout and Acyl-CoA: cholesterol acyltransferase 1 in knockout mice, the study finds that myocardial infarction significantly reduces irisin levels, contributing to muscle degradation and oxidative stress [151]. However, aerobic exercise partially reverses these effects by upregulating Irisin, suggesting its crucial role in enhancing muscle resilience and reducing apoptosis. This highlights irisin’s potential as a therapeutic target for mitigating skeletal muscle atrophy in heart failure conditions [151].

Currently, there are no specific human clinical trials that focus exclusively on irisin’s effectiveness in treating oxidative stress-induced CVDs. However, research indicates that irisin, a myokine induced by exercise, holds potential benefits in this area due to its physiological functions, such as reducing inflammation and improving metabolic profiles. Studies have shown that irisin can play a positive role in cardiovascular health by promoting white fat browning, enhancing metabolism, and alleviating inflammation [152]. These effects suggest that irisin could potentially help in managing oxidative stress and its related cardiovascular complications [153]. However, more clinical research is needed to determine irisin’s therapeutic potential in human.

#### 6.1.5. Cannabinoids

The role of cannabinoids in oxidative stress and cardiovascular disease is an area of ongoing research. Cannabinoids, the active compounds found in cannabis, have been shown to exert complex effects on the cardiovascular system. Some studies suggest that cannabinoids, particularly cannabidiol (CBD), may have antioxidant properties, potentially reducing oxidative stress in the cardiovascular system [154,155]. It is due to the fact that CBD interacts with the endocannabinoid system and influences oxidative stress regulation through various receptors, such as cannabinoid receptors (CB1 and CB2), ionotropic receptors (TRP), and nuclear receptors (PPAR) [156]. Increased production of ROS and activation of NOXs in atherosclerosis correlate with elevated 2-AG biosynthesis in the vessel wall, potentially indicating a compensatory response to oxidative stress through CB2 signaling [157].

CBD has shown potential benefits in reducing oxidative stress and inflammation, which are critical factors in the development of CVDs. Its interaction with CB2 receptors, in particular, is associated with anti-inflammatory and antioxidative effects that may protect against vascular damage and atherosclerosis [154]. Additionally, cannabinoids may influence the expression of antioxidant enzymes and modulate pathways involved in cell survival and apoptosis, further contributing to their potential protective effects on the cardiovascular system [158].

However, studies on the beneficial effects of cannabinoids are still ongoing and controversial. While preclinical studies provide promising results, clinical trials are necessary to fully understand the therapeutic potential and safety of cannabinoids in managing oxidative stress and CVDs. The complexity of cannabinoid interactions with various receptors and signaling pathways warrants further investigation to elucidate their exact mechanisms and therapeutic applications.

### 6.2. Nanoparticle Therapies

#### 6.2.1. Polyoxalate-Based Targeted Nanoparticles

Targeted nanoparticles to the areas of oxidative stress have been shown to effectively treat ROS-related diseases. These nanoparticles contain H_2_O_2_-responsive peroxalate ester linkage that rapidly degrades at the site of high levels of H_2_O_2_, which then releases vanillyl alcohol (VA) that exerts anti-inflammatory and anti-apoptotic activities [159]. In various animal models of I/R injuries, such as acute hepatic and cardiac I/R injuries, these nanoparticles demonstrated potent anti-inflammatory and anti-apoptotic activities resulting in reduced organ damage [160,161]. They also significantly increased survival outcome in doxorubicin-induced cardiac and hepatic toxicities in vivo through decreasing oxidative stress injuries [162]. Furthermore, in a rat model of whole-body ischemia/reperfusion injury, these nanoparticles prevented critical organ damage by their antioxidative, anti-inflammatory, and anti-apoptotic effects [163]. In a murine I/R injury model, incorporating neuropeptide Y3-36 into these nanoparticles also enhanced angiogenesis, and significantly reduced infarct size and mortality [164].

#### 6.2.2. Nanomicelle Delivery System for Curcumin

As mentioned before, curcumin is one of the components of turmeric and possesses anti-inflammatory and antioxidative features [128]. To more effectively deliver curcumin, curcumin nanomicelle capsules were generated as a novel formulation which was designed to increases the bioavailability of curcumin [165]. With increased oral absorption due to intact soft gel passage through the stomach to the small intestine [166], studies demonstrated that curcumin nanomicelle capsules can be used to significantly reduce cardiovascular events occurring due to oxidative stress [167,168]. Mogharrabi et al. performed a clinical trial involving 70 CAD patients with curcumin and showed that curcumin nanomicelle significantly reduced MMP-2 and MMP-9 activities and gene expression compared to a placebo, suggesting it as a novel treatment in secondary prevention of cardiovascular events [169]. Also, Helli et al. supported this in the clinical trial involving patients undergoing coronary angioplasty, where both curcumin and nano-curcumin led to significant changes in total antioxidant capacity, and malondialdehyde (MDA) levels, suggesting its potential as a more effective antioxidant therapeutic option for cardiac patients due to its higher bioavailability [170].

## 7. Biomarkers for Oxidative Stress Assessment

### 7.1. Soluble Transferrin Receptor

Soluble transferrin receptor (sTfR), a cellular membrane protein, reflects the iron demand of the body and is known to be influenced by body iron stores and erythropoietic activity [171]. The study’s findings highlight a significant positive correlation between sTfR and total antioxidant capacity across both genders, suggesting a potential role of sTfR in enhancing the body’s antioxidant defense. Additionally, it shows that higher sTfR levels are positively associated with increased waist circumference and fasting glucose levels, among other cardiometabolic risk factors [172]. The study highlights sTfR as a potentially useful biomarker for evaluating antioxidant status and cardiometabolic risk in healthy individuals [173]. It suggests that higher sTfR levels are associated with better antioxidant capacity and certain cardiometabolic parameters, independent of other iron markers like ferritin and hepcidin.

### 7.2. Transthyretin (TTR)

Transthyretin amyloid cardiomyopathy (ATTR-CM) is a rare but serious condition, leading to heart failure or conduction system problems [174]. ATTR amyloidosis is caused by TTR misfolding [175], leading to the progressive and potentially fatal cardiomyopathy caused by extracellular deposition of transthyretin-derived insoluble amyloid fibrils in the myocardium [176]. Current studies have shown that the lower TTR concentration has been linked to obesity and diabetes [177], which are the common comorbidities for heart failure. Furthermore, the toxic TTR oligomers can lead to significant tissue injury and an inflammatory response [178].

In the previous studies of ATTR-CM, TTR misfolding has been linked to various biological processes involving oxidant and antioxidant properties, making it a potential biomarker or therapeutic target [179]. There is a strong correlation between TTR levels and the generation of superoxide radicals and nitrate and nitrite ions, emphasizing the need to determine TTR oligomer levels to assess the extent of oxidative stress [179]. TTR can also induce oxidative stress in endoplasmic stress (ER) and hence involved in the unfolded protein response [180]. Consequently, the function of TTR recommends mitochondrial antioxidants for treatment [181], called TTR stabilizers, such as quercetin, epigallocatechin gallate (EGCG), gallic acid, curcumin, and propolis extract [182]. The treatment for TTR-related cardiac amyloidosis usually involves slowing fibril production and deposition, including gene silencing, TTR stabilization, and the destruction and re-absorption of amyloid deposits [183].

### 7.3. Cystatin C

Cystatin C is a protein that inhibits cysteine proteases and is produced by all nucleated cells [184]. A study has highlighted Cystatin C in treating MI/RI, a condition often occurring after a heart attack, where tissue damage results from the restoration of blood supply [185]. Also, Cystatin C is a novel biomarker to identify renal dysfunction and cardiovascular risk [186].

## 8. Discussion and Conclusions

The findings of this review highlight the multifaceted role of antioxidants in health and disease management. Our analysis suggests that while antioxidants offer significant protective effects against oxidative stress and related pathologies, their efficacy can vary greatly depending on dosage, source, and individual health status. This aligns with and expands upon the existing literature, indicating a need for a more nuanced understanding of antioxidant interactions within the human body. However, it is vital to acknowledge the limitations of our review, particularly in the context of differing methodologies and scopes of the studies analyzed. This variability underscores the complexity of antioxidant research and the challenges in drawing definitive conclusions.

In conclusion, this review underscores the potential of antioxidants as a key component in preventing and managing oxidative stress-related conditions. However, it also highlights the complexity and variability inherent in antioxidant research. Future studies should standardize methodologies and focus on long-term, comprehensive analyses to better understand the role of antioxidants in human health. The findings from this review contribute to a growing body of knowledge, reinforcing the importance of antioxidants while calling for a more detailed and nuanced approach to their study and application in healthcare.
